# Pre-exposure to Lower-Level Noise Mitigates Cochlear Synaptic Loss Induced by High-Level Noise

**DOI:** 10.3389/fnsys.2020.00025

**Published:** 2020-05-12

**Authors:** Liqiang Fan, Zhen Zhang, Hui Wang, Chunyan Li, Yazhi Xing, Shankai Yin, Zhengnong Chen, Jian Wang

**Affiliations:** ^1^Department of Otolaryngology-Head and Neck Surgery, Shanghai Jiao Tong University Affiliated Sixth People’s Hospital, Shanghai, China; ^2^Otolaryngology Institute of Shanghai Jiao Tong University, Shanghai, China; ^3^Shanghai Key Laboratory of Sleep Disordered Breathing, Shanghai, China; ^4^School of Communication Sciences and Disorders, Faculty of Health, Dalhousie University, Halifax, NS, Canada

**Keywords:** noise exposure, synaptic loss, coding-in-noise deficits, Guinea pigs, toughening, conditioning, priming

## Abstract

The auditory sensory organs appear to be less damaged by exposure to high-level noise that is presented after exposure to non-traumatizing low-level noise. This phenomenon is known as the toughening or conditioning effect. Functionally, it is manifested by a reduced threshold shift, and morphologically by a reduced hair cell loss. However, it remains unclear whether prior exposure to toughening noise can mitigate the synaptic loss induced by exposure to damaging noise. Since the cochlear afferent synapse between the inner hair cells and primary auditory neurons has been identified as a novel site involved in noise-induced cochlear damage, we were interested in assessing whether this synapse can be toughened. In the present study, the synaptic loss was induced by a damaging noise exposure (106 dB SPL) and compared across Guinea pigs who had and had not been previously exposed to a toughening noise (85 dB SPL). Results revealed that the toughening noise heavily reduced the synaptic loss observed 1 day after exposure to the damaging noise. Although it was significant, the protective effect of the toughening noise on permanent synaptic loss was much smaller. Compared with cases in the control group without noise exposure, coding deficits were seen in both toughened groups, as reflected in the compound action potential (CAP) by signals with amplitude modulation. In general, the pre-exposure to the toughening noise resulted in a significantly reduced synaptic loss by the high-level noise. However, this morphological protection was not accompanied by a robust functional benefit.

## Introduction

Pre-exposure to a continuous low-level noise has been said, according to a large number of previous reports, to reduce the hearing loss caused by the subsequent high-level noise exposure (Canlon et al., 1988; Ryan et al., 1994; Pukkila et al., 1997; Attanasio et al., 1998; Ahroon and Hamernik, 1999; Hamernik and Ahroon, 1999a; Alvarado et al., 2016). This protective effect exerted by the low-level noise is termed a toughening, priming or conditioning effect. This phenomenon is functionally demonstrated by a reduced threshold shift, and morphologically by reduced hair cell loss. While the toughening phenomenon has been widely observed in several animal species, including Guinea pigs, mice, chinchillas, rats (Canlon et al., 1988; Pukkila et al., 1997; Hamernik et al., 1998; Qiu et al., 2007; Alvarado et al., 2016) and human beings (Cowan, 1983; Inaoka et al., 1992; Miyakita et al., 1992; Attanasio et al., 1998; Niu and Canlon, 2002b), the molecular mechanisms underlying the toughening effect have only been examined in a few reports, which failed to yield a clear conclusion.

Hair cells, especially outer hair cells (OHCs) and their surrounding structures, have been recognized as the major targets in noise-induced cochlear damage (Canlon, 1988; Rajan, 2001; Hu et al., 2002; Nicotera et al., 2003; Bohne et al., 2007; Park et al., 2014; Goutman et al., 2015). Damage and loss of OHCs are the major mechanisms underlying the noise-induced threshold shift (Hamernik et al., 1998; Hamernik and Qiu, 2000; Rajan, 2001). Therefore, OHCs are the major focus in the study of noise-induced hearing loss. More recently, the synapse between inner hair cells (IHCs) and type I spiral ganglion neurons (SGNs) has been identified as another locus of noise-induced cochlear damage. Substantial synaptic loss can be induced by exposure to a single, brief noise sufficiently mild such as not to result in a permanent threshold shift (e.g., 100 dB SPL 2 h in mice (Kujawa and Liberman, 2009) and 106 dB SPL 2 h in rats (Furman et al., 2013) and Guinea pigs (Lin et al., 2011; Liu et al., 2012; Shi et al., 2016; Song et al., 2016). Functionally, the damage to the synapse could exert a greater impact on hearing functions, even when the hearing threshold remains unchanged. First, the synapses innervating a special group of auditory never fibers are prone to noise damage (Furman et al., 2013; Song et al., 2016). This group of auditory never fibers has a high threshold, larger dynamic range and is therefore critical for coding sound at higher intensity levels and against a background noise (Liberman, 1978, 1980; Liberman and Kiang, 1984; Tsuji and Liberman, 1997; Taberner and Liberman, 2005; Liberman et al., 2011). Second, the interrupted synapses can be partially re-established (Shi et al., 2013, 2015a; Kaur et al., 2019; Kim et al., 2019), but the repaired synapses have been found to have coding deficits (Shi et al., 2015a, 2016; Song et al., 2016; Chen et al., 2019a). Since the noise-induced synaptic damage, or synaptopathy, can be established by noise without causing hearing loss defined by a threshold shift *per se*, noise-induced hidden hearing loss has been used as an umbrella term to reflect the functional deficits at suprathreshold levels (Moser and Starr, 2016; Plack et al., 2016; Song et al., 2016; Kobel et al., 2017; Liberman and Kujawa, 2017; Liberman, 2017; Lobarinas et al., 2017; Chen et al., 2019a).

While the protective effect of a toughening noise has been demonstrated to correspond to reducing the noise damage and death of OHCs, no evidence has yet pointed to whether pre-exposure to a low-level noise can reduce the synaptic loss caused by a subsequently traumatizing noise. In the present study, synaptic loss caused by a 2-h exposure to noise at 106 dB SPL was compared between two groups, one of which consisted of animals pre-exposed to a low-level noise at 85 dB SPL, 8 h/day for 3 days. The results revealed that the toughening effect is associated with the noise-induced synaptic loss in Guinea pigs.

## Materials and Methods

### Subjects and General Procedures

**Figure 1:**
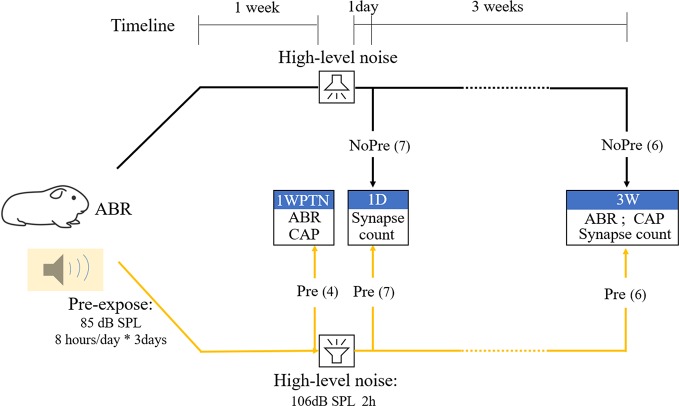
Flowchart representing the main procedures and subject grouping in the experiment. CTL, control group; NoPre, group without pre-exposed; Pre, group with pre-exposed; 1WPTN, 1-week post-toughening noise; 1D, 1-day post-high-level noise; 3W, 3 weeks post-high-level noise. The numbers in parentheses represent the sample sizes of each group.

Thirty-seven male albino Hartley Guinea pigs (2-months-old) were obtained from the Shanghai Songlian Lab Animal Field (Shanghai, China) for this study after they passed an otoscopic examination (to rule out middle ear infection) and auditory brainstem response (ABR) threshold test (to ensure normal cochlear sensitivity). They were divided randomly into three groups: the pre-exposure group (*n* = 17), exposed to a toughening noise before a traumatizing noise, the group without pre-exposure (*n* = 13), exposed to a traumatizing noise only, and the control group (CTL, *n* = 7), not exposed to any noise. Figure 1 represents the flow chart representing the major procedures in the experiment. After a baseline ABR test, guinea pigs in the pre-exposure (Pre) group were exposed to the toughening noise. One week later, experiments in four of them (labeled as the subgroup of Pre-1WPTN) from the pre-exposed group ended after the tests of ABR and compound action potential (CAP). The remaining subjects in the Pre group and all subjects in the group without pre-exposed (NoPre group) were exposed to the traumatizing high-level noise 1 week after the pre-expose. One day post-high-level noise exposure, seven subjects in each of the pre-exposed group and the no pre-exposed group were sacrificed for synapse counting. These subgroups were labeled Pre-1D and NoPre-1D, respectively. The remaining six animals in each of the pre-exposed group and no pre-exposed group groups were tested with ABR and CAP tests before being sacrificed for synapse counting at 3 weeks post-high-level noise exposure (corresponding to the subgroups labeled Pre-3W and NoPre-3W, respectively). The seven subjects in the CTL were also examined using similar methods at this time.

All procedures were approved by the Institutional Animal Care and Use Committee (IACUC) of the Affiliated Sixth People’s Hospital, Shanghai Jiao Tong University (permit number DWLL2017-0295).

### Noise Exposure

The noise used to induce synaptic damage consisted of white noise, high pass filtered at 4 kHz, and presented for 2 h at 106 dB SPL. The noise used in the toughening procedure consisted of white noise, high pass filtered at 4 kHz, and presented at 85 dB SPL, 8 h/day for three consecutive days (yielding 24 h of exposure). The upper-frequency limit of the noise was 22 kHz due to the frequency responses of the speakers (Pyramid TW67 Super tweeters, Pyramid, Brooklyn, NY, USA). During the noise exposure, the animals were awake, and unrestrained in metal wire cages, on a floor placed 40 cm below a four-speaker array. The noise level was monitored throughout the exposure.

### Electrophysiological Evaluation

All electrophysiological evaluations were performed in an electromagnetically shielded sound booth. A mixture of ketamine and xylazine was used for anesthesia of the Guinea pigs for the auditory responses and was administrated by intraperitoneal injection. The initial dose was 40 and 10 mg/kg for ketamine and xylazine, respectively, and $\frac{1}{4}$ of the initial dose was added each hour until the end of the test, as needed. The ABR was recorded with three subdermal electrodes, with the recording electrode inserted at the vertex and the reference and grounding electrodes positioned posterior to the external auditory canals. The CAP was recorded with a silver wire electrode that was placed on the round window membrane after the mastoid was surgically opened. The reference and ground electrodes were the same for the CAP and ABR tests. The biological signals picked up by the electrodes were led to a RA4PA preamplifier from Tucker-Davis Technologies (TDT System III; Alachua, FL, USA).

The stimulus generation and bio-signal acquisition parameters were similar to those used in our previous study (Chen et al., 2019b). Briefly, the acoustic stimuli included: (1) clicks for CAP: 0.1 ms duration, presented at 21.1/s; (2) tone bursts (TB) for ABR and CAP: 10 ms duration and 0.5 ms rise/fall time presented at 21.1/s; and (3) amplitude modulation (AM) tones for AM CAP: 500 ms duration and a rise/fall time of 5 ms with a carrier frequency of 16 kHz, modulation frequencies (modulation frequency) of both 93 and 675 Hz, and modulation depths of 30% and 100% at each modulation frequency. Stimuli (1) and (2) were presented from 90 to 0 dB SPL in decreasing steps of 5 or 10 dB. When testing AM responses, a stimulus (3) was presented at 80 dB SPL. The masker consisted of white noise, high-passed filtered at 4 kHz, and presented at a signal-noise-ratio (SNR) of 0 dB. The stimuli and masker were played out separately *via* two broadband speakers (MF1; TDT, USA) that were placed 10 cm in front of the animal’s head.

Evoked responses were amplified 20 times by a PA4 preamplifier (TDT) and averaged 1,000 times for the ABRs, 100 times for the click and tone burst CAPs, and 50 times for AM CAPs. The ABR threshold was defined as the lowest level at which a repeatable wave III was observed and was tested at all frequencies from 1 to 32 kHz. The CAP amplitude (in response to the click and the TB) was defined as the difference between the first negative peak (N_1_) and the following positive peak (P_1_). To analyze the AM CAP, a centered 400-ms portion of the response to the 500-ms AM sweep was subjected to a spectrum analysis (*via* fast Fourier transformation). Peak values corresponding to the modulation frequencies were measured from the amplitude spectrum of the responses as an estimate of the strength of AM CAP phase locking.

### Morphology

After the final physiological tests, the animals were sacrificed, and their cochleae were used for quantifying the synaptic ribbons, based on the methods of our previously published protocols (Liu et al., 2012; Shi et al., 2013; Song et al., 2016). Briefly, after fixing with 4% paraformaldehyde in phosphate-buffered saline (PBS), the cochlear tissues were dissected, permeabilized with 1% Triton X-100 in PBS for 1 h, incubated in 5% goat serum in PBS for another hour, and incubated overnight at 4°C with primary antibodies against C-terminal binding protein 2 (CtBP2; mouse IgG1 to CtBP2; BD Biosciences, Franklin Lakes, NJ, USA: cat. # 612044, 1:200). After the reaction, the tissues were washed and treated with a secondary antibody (A21124, Invitrogen, Carlsbad, CA, USA) for 2 h at room temperature, and mounted on microscope slides. Confocal images were acquired at specified frequency positions based on frequency-distance mapping (Viberg and Canlon, 2004) using a confocal laser-scanning microscope (LSM 710 META; Zeiss, Shanghai, China) with a 63× water-immersion objective. Z-stack images were taken with a step size of 0.2 mm to cover the entire synaptic pole of the hair cells from the site of the inner spiral bundle to the ribbons in the supranuclear region. The stack images were then exported to ImageJ image-processing software (National Institutes of Health, Bethesda, MD, USA) for automatic identification of puncta, and the synapse density (synapse#/IHC) was calculated across multiple frequency points to generate a synapse density cochleogram. At each frequency, the total number of CtBP2 puncta were counted from 10 to 20 IHCs and the average density was calculated.

### Statistics

All data are expressed as means ± standard error of the mean (SEM). ANOVAs followed by *post hoc* testing (Tukey’s method) were performed using SigmaPlot (ver.14; Systat Software Inc., San Jose, CA, USA). In all analyses, *p* < 0.05 was considered indicative of statistical significance.

## Results

### ABR Threshold

**Figure 2:**
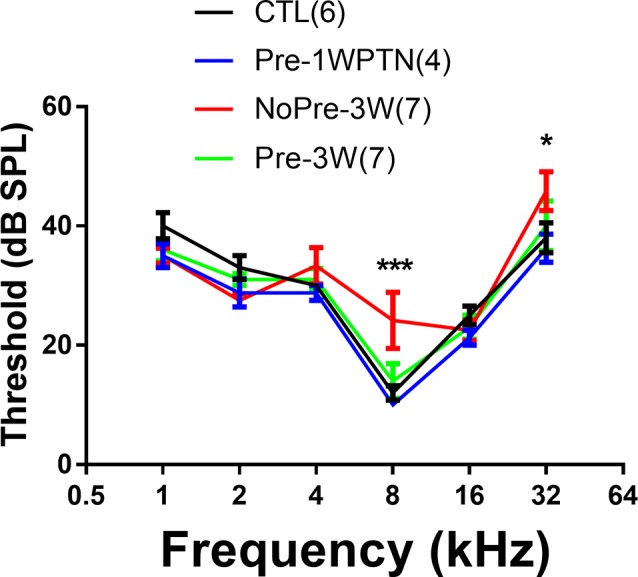
Auditory brainstem response (ABR) threshold audiograms: a comparison across the four subgroups. The sample size of each group is listed in parentheses. The thresholds were significantly higher in the Pre-1WPTN than the control group at 8 and 32 kHz. The number of asterisks indicates the significance level (***p < 0.001, *p < 0.05) of the *post hoc* pairwise comparison against the control group within each frequency after the two-way ANOVA against the factors of group and frequency.

ABR thresholds were compared between the CTL and three subgroups in noise-treated animals, including the Pre-1WPTN, NoPre-3W, and Pre-3W (Figure 2). A significant group effect was seen in a two-way ANOVA against the factors of group and frequency (*F*_(3,96)_ = 3.682, *p* = 0.015). Within each frequency, only the thresholds of the Pre-1WPTN group were significantly higher than the control values at 8 and 32 kHz (*post hoc* test, Tukey’s method, *q* = 5.722, *p* < 0.001, at 8 k; *q* = 3.87, *p* = 0.037 at 32 k). There was no significant difference between the CTL and either the NoPre-3W or Pre-3W. The number followed by the group name was the number of animals in the group.

### Noise-Induced Synaptic Loss

**Figure 3:**
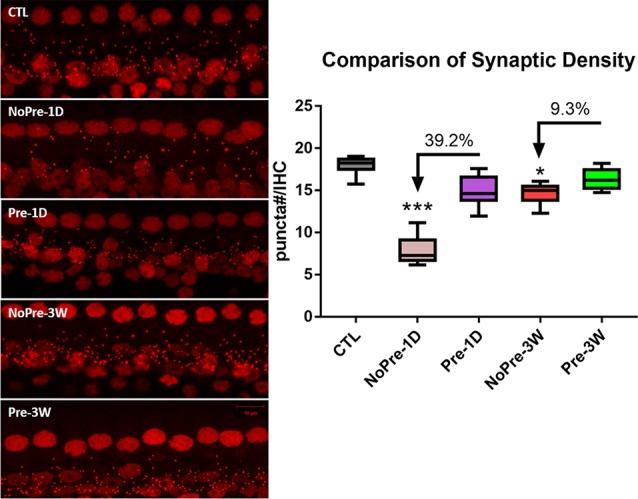
Comparison of synapse counts across groups. Left: representative images of cochlear surface preparation showing presynaptic puncta stained with a CtBP2 antibody. The ribbon puncta were shown as the small red dots. All images were taken from 16 kHz regions. Right: the comparison of the synaptic density averaged across 8–32 kHz regions. The number of ribbon puncta represents the number of synapses, and the synaptic density was calculated as the # puncta/IHC. CTL, control group; 1D, 1-day post-high-level noise; 3W, 3 weeks post-high-level noise; NoPre, group without pre-exposed; Pre, the group with pre-exposed; The between-group comparison was done at 1 day and 3 weeks after the damaging noise, for the evaluation of both temporary and permanent synaptic loss respectively, by using *post hoc* pairwise comparison (Tukey method) after one-way ANOVA. The number of asterisks represents the level of significance: ***p < 0.001, *p < 0.05.

**Figure 4:**
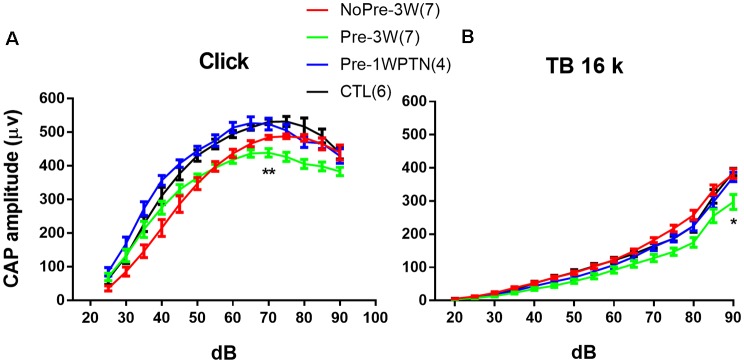
Compound action potential (CAP) input/output (IO) curves. **(A)** clicks and **(B)** 16 k tone bursts. CTL, control group; Pre-1WPTN, 1-week post-toughening noise group; 3W, 3 weeks post-high-level noise; NoPre, group without pre-exposed; Pre, group with pre-exposed. The number of asterisks represents the level of significance (*p < 0.05, **p < 0.01) for the *post hoc* pairwise tests (Tukey method) against the control group after the two-way ANOVA on the factors of group and level.

Figure 3 shows typical images from 16 kHz of the sensorial epithelia of the cochleae from each group. The surface preparation was stained with an antibody against CtBP2 to show the puncta of the presynaptic ribbons. A large decrease in the number of ribbons was evident 1 day after exposure to high-level noise, especially in animals that were untoughened (NoPre-1D). The images taken 3 weeks after exposure to a high-level noise show that the synapse count had largely returned to its control value, and it is difficult to detect any difference between the two noise groups based on a single image comparison (NoPre-3W and Pre-3W). Figure 4 summarizes the synaptic density (# CtBP2 puncta/IHC) assessed across different groups and at different time points after the noise treatment. Our analysis was focused on the three high-frequency spots (8, 16 and 32 kHz) due to the high susceptibility of synapses in these regions of the cochlea (Liu et al., 2012; Shi et al., 2013; Song et al., 2016). The average ribbon density across the three frequency regions was 18 ± 0.258 ribbons/IHC in the CTL. This number was only 7.89 ± 0.409 ribbons/IHC at NoPre-1D, yielding an average 56.2% drop from the control value. In contrast, synaptic loss at this time point, named temporary synaptic loss, was only 16.9% in the Pre-1D (14.967 ± 0.460 ribbons/IHC), resulting in a large difference of 39.3% between the two groups (Figure 3). A larger recovery in synapse count was seen in the NoPre-3W thereafter. However, the synaptic loss counted at 3 weeks after high-level noise, termed permanent loss, was still larger in the NoPre-3W (18.7%) than in the Pre-3W (9.4%), although the between-group difference was 9.3%, much smaller than the difference in temporary loss (Figure 3). A significant overall difference was revealed by a one-way ANOVA (*F*_(4,57)_ = 113.817, *p* < 0.001). *Post hoc* pairwise comparisons showed that the synaptic density observed at 1 day after high-level noise exposure for both Pre- and NoPre groups were significantly lower than the CTL (Tukey method, *q* = 8.343, and 28.368 for both Pre-1D and NoPre-1D respectively, *p* < 0.001), suggesting a significant temporary loss in both groups. Besides, the density observed 3 weeks after the damaging noise were also significantly lower than that of the CTL (Tukey method, for Pre-3W: *q* = 4.200, *p* = 0.034; and for NoPre-3W: *q* = 9.070, *p* < 0.001), suggesting a significantly permanent synaptic loss in both groups. Most importantly, however, there was also a significant difference between the two noise groups (NoPre-1D vs. Pre-1D: *q* = 19.495, *p* < 0.001). Furthermore, permanent loss was also significantly lower in the pre-exposure group at 3 weeks post-high-level noise exposure (Pre-3W vs. NoPre-3W: *q* = 4.023, *p* = 0.047), although the difference was much smaller than of the difference in temporary loss. Data used in this analysis were shown as Group (“number of animals,” “number of ears”) for Pre-1D (7, 13), Pre-3W (6, 9), NoPre-1D (7, 14), NoPre-3W (6, 12), CTL (7, 14), respectively.

### The Impact of Noise Exposure on Cochlear Output

Figure 4 represents the comparison of the input/output (I/O) functions of CAP evoked by both clicks and tone bursts at 16 kHz. Data used in the analysis showed as Group (“number of animals,” “number of ears”): Pre-1WPTN (4, 8), Pre-3W (5, 9), NoPre-3W (5, 9), CTL (5, 6).

Overall, the click CAP IO curves were largely overlapping between the CTL and Pre-1WPTN, while the 16 k TB CAP IO curves were largely overlapping across the CTL, Pre-1WPTN, and NoPre-3W. However, the overall amplitude of the CAP IO curve was lowest in the Pre-3W. A two-way ANOVA against the factors of grouping and sound level revealed a significant effect of grouping (*F*_(3,392)_ = 57.493, *p* < 0.001 for click CAP and *F*_(3,392)_ = 43.917, *p* < 0.001 for 16 k TB CAP). *Post hoc* tests (Tukey’s method) revealed a significant difference in the maximal click CAP between the Pre-3W and CTL (*q* = 4.990, *p* = 0.002), which was examined at 70 dB SPL. However, no significant difference was observed in the maximal CAP amplitude between the CTL and NoPre-3W. In the 16k TB CAP, the maximal amplitude of the Pre-3W was also significantly lower than that of the CTL (*q* = 8.091, *p* < 0.001), while there was no significant difference across the other three subgroups (Ctrl, Pre-1WPTN, NoPre-3W).

### The Impact of Noise Exposure on the AM CAP

The AM CAP was assessed with a 16 kHz carrier at 80 dB SPL and modulated using two modulation depths (30% and 100% respectively) at each of the modulation frequencies (93 and 675 Hz). Since the CAP was recorded with the round window electrode that was placed during open-ear surgery, the stability of the electrode was a concern. To prevent any potential complications associated with electrode instability, CAP responses were sequentially tested in quiet, masked, and quiet conditions. If CAP amplitude differences between the two tests in the quiet condition were larger than 5 dB, the data were not used. The results of the two tests in the quiet condition were averaged to calculate the masking effect.

**Figure 5:**
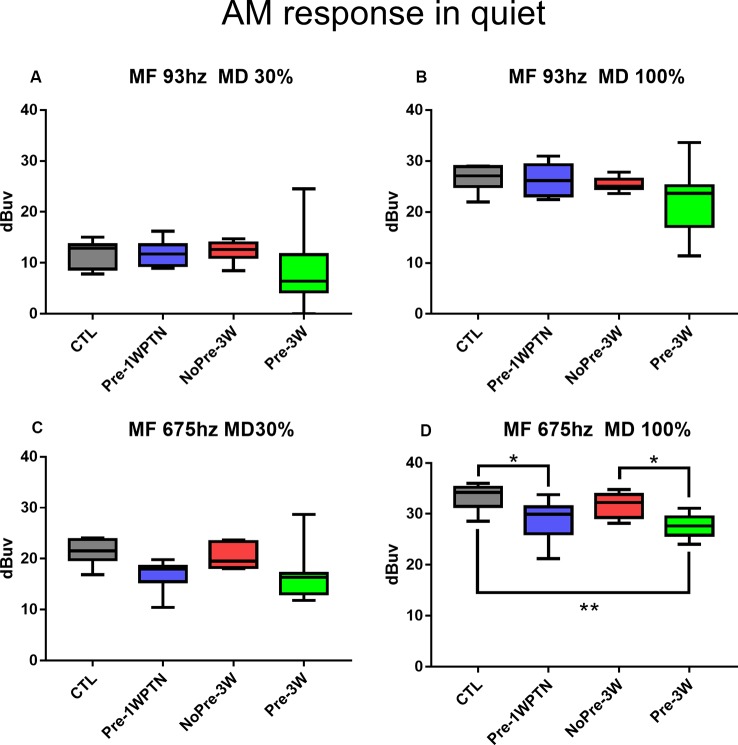
Comparison of AM CAP amplitudes in the quiet condition. **(A–D)** AM CAP obtained at different modulation frequencies and depths indicated in the panel titles. CTL, control group; Pre-1WPTN, 1week post-toughening noise group; 3W, 3 weeks post-high-level noise; NoPre, group without pre-exposed; Pre, group with pre-exposed. The number of asterisks shows the significant level o (*p < 0.05, **p < 0.01) of the *post hoc* tests (Tukey method) after one-way ANOVA.

Figure 5 summarizes the results of the AM CAP tests in the quiet condition across the four groups. Overall, the CAP amplitude was smaller in the toughened group (TG) compared with the UTG at 3 weeks post-HN. However, a significant group difference was only seen at a modulation frequency of 675 Hz and a modulation depth of 100% (Figure 5D), which was examined under this condition by a one-way ANOVA to assess the group effect (F_3, 28_ = 6.384, *p* = 0.002). A significant difference in AM CAP amplitude was found between the Pre-1WPTN and CTL (Tukey test, *q* = 4.171, *p* = 0.031), between the TG and UTG at 3WPHN (*q* = 4.267, *p* = 0.026), and between the Pre-3W and CTL (*q* = 5.462, *p* = 0.003).

**Figure 6:**
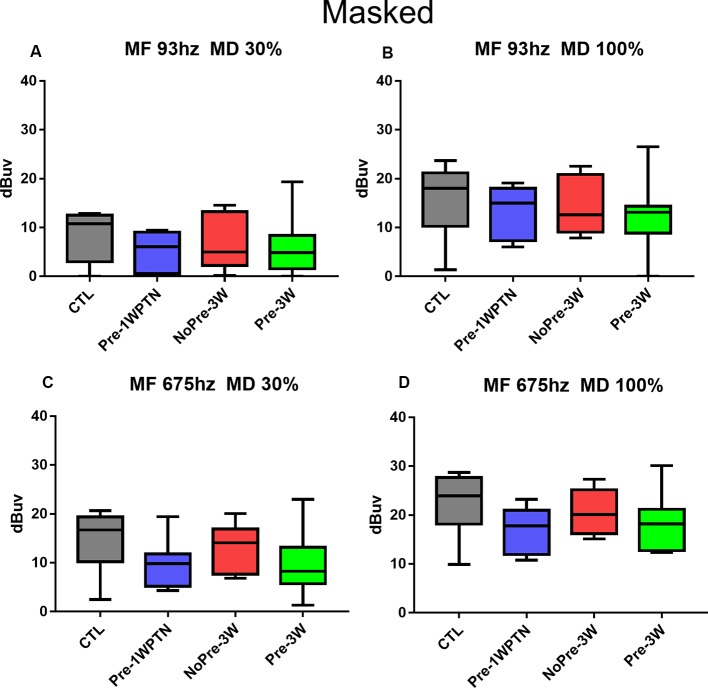
Comparison of AM CAP amplitude across groups when masking the HP noise. **(A–D)** AM CAP obtained at different modulation frequencies and depths indicated in the panel titles. CTL, control group; Pre-1WPTN, 1week post-toughening noise group; 3W, 3 weeks post-high-level noise; NoPre, group without pre-exposed; Pre, group with pre-exposed.

Figure 6 summarizes the results of the AM CAP test when masking the HP (>4 kHz) noise across the four groups (CTL, Pre-1WPTN, NoPre-3W, and Pre-3W). No significant difference was seen across the groups in any combination of modulation depth s and modulation frequencies.

## Discussion

In the present study, prior exposure to continuous noise at 85 dB SPL produced a toughening effect in Guinea pigs, reducing the synaptic loss incurred by exposure to high-level noise. Similar to our previous studies (Shi et al., 2013, 2015b; Song et al., 2016), exposure to a traumatizing noise at 106 dB SPL for 2 h produces a temporary synaptic loss of ~50% in the untoughened group. However, this was heavily reduced in the toughened group to only 16.9% on average. Although it was much smaller than the change in temporary synaptic loss, the mitigation by the toughening noise on the permanent synaptic loss, which was observed 3 weeks after the exposure to the traumatizing noise, remained significant (Figure 3). In CAP tests 3 weeks after the traumatizing noise, the animals in the toughened group were slightly disadvantaged in terms of both maximal CAP amplitude in response to clicks and 16 kHz tone bursts (Figure 4), and in AM CAP amplitude in the quiet condition (Figure 5). No significant difference was seen in terms of the masked AM CAP between any of the groups (Figure 6).

The toughening effect of “non-damaging” noise, which is also termed conditioning or priming effect, is well-known in the study of noise-induced hearing loss since the earliest reports were published around 1990 (Canlon et al., 1988; Subramaniam et al., 1991, 1996; Henderson and Subramaniam, 1993; Henselman et al., 1994; Pukkila et al., 1997). The reduced threshold elevation and hair cell death incurred by prior exposure to such noise has been demonstrated in different experimental animals, including rats (Pukkila et al., 1997), Guinea pigs (Canlon et al., 1988; Attanasio et al., 1999), chinchillas (Subramaniam et al., 1991; Hamernik et al., 1998, 2003; Jacono et al., 1998; Ahroon and Hamernik, 1999; Hamernik and Ahroon, 1999b; Qiu et al., 2007), and mice (Tahera et al., 2007). The toughening effect has been observed when continuous, interrupted and even impulse noises (Henselman et al., 1994; Skellett et al., 1998; Ahroon and Hamernik, 1999) are used as the toughening noise. In the present study, the protective effect on reducing noise-induced synaptic loss was demonstrated by prior exposure to a continuous noise in Guinea pigs. The study should be extended to other types of acoustic conditioning and species to further confirm the content of the toughening effect on reducing noise-induced synaptic loss.

It is interesting to see that the protective effect on permanent synaptic loss is much smaller than that on temporary synaptic loss. This discrepancy suggests that the toughened synapses are less likely to be repaired whenever they are destroyed. Also, the functional disadvantages in the toughened group reveal the limitation of the protective effect by toughening. One possibility is that the toughening noise is not a “non-damaging” noise. The toughening noise at 85 dB SPL for 8 h per day is at the upper limits of current safety standards with regard to noise-induced hearing loss. Based upon available data, such noise exposure would not cause OHC damage and therefore no permanent threshold shift (Kujawa and Liberman, 1999). Therefore, such toughening protocol could be used in humans such as those warfighters to mitigate synaptic damage on the battlefield. In the present study, we did not assess the potential synaptic loss by the toughening noise because a previous study reported no significant synaptic loss after a similar exposure in mice (Maison et al., 2013). However, it is possible that minor damage has been incurred around the synapses, or that some synapses may have experienced severe damage but have been repaired. Such possibilities may be responsible for the functional disadvantages seen in the TG.

In the present study, synapses were counted only based on the puncta of presynaptic ribbons, not by those of post-synaptic terminals. This method is supported by our previous studies in which the change in ribbon counts was found to be very similar to the change in post-synaptic terminals stained by an antibody against post-synaptic densities (Liu et al., 2012; Shi et al., 2013, 2015a). Such a high correlation was also reported by other studies in which the post-synaptic terminal was labeled by an antibody against glutamate receptors (Liberman et al., 2015; Sebe et al., 2017; Kim et al., 2019).

The mechanisms of the protective effect of the toughening noise are not entirely clear, although several hypotheses have been assessed in previous studies. Many previous studies have established a strong link between the toughening effect and the regulation of antioxidative activity in the auditory system. Oxidative stress is largely increased by exposure to a traumatizing noise and is considered a major reason for the hair cell death induced by acoustic overstimulation (see reviews, Le Prell et al., 2003; Henderson et al., 2006; Choi and Choi, 2015; Waqas et al., 2018; Ye et al., 2018; Hahad et al., 2019). Pre-exposure to a toughening noise was reported to increase the level of catalase in the stria vascularis of chinchillas (Jacono et al., 1998). The protective effect of the toughening noise is likely due to the clearance of hydrogen peroxide (·OH) and maintenance of low-level glutathione in the cochlea by this scavenging enzyme (Harris et al., 2006). Furthermore, the acoustic conditioning effect on HCs was proven to depend on the activation of the hypothalamic-pituitary-adrenal axis, which resulted in up-regulated plasma corticosterone and glucocorticoid receptors in the cochlea (Tahera et al., 2007). The critical role of this up-regulation was demonstrated by the loss of this protective effect in mice who had previously undergone adrenalectomy or in whom a blocking agent was used (Tahera et al., 2007). More recently, the protective effect of a toughening noise was again linked to its potential anti-oxidant effect by the increase in the calcium buffering capacity of the cochlea of rats (Alvarado et al., 2016).

While the antioxidant effect of the toughening noise is well supported in the context of the reduced damage to OHCs, which are responsible for the reduction in the noise-induced threshold shift, it remains unclear whether this mechanism can fully account for the reduced synaptic loss observed in the present study. It is well-known that noise-induced synaptic damage is mediated by glutamic excitotoxicity (Puel et al., 1998; Pujol and Puel, 1999; Hakuba et al., 2000). In a recent study, this excitotoxicity on cochlear ribbon synapses was found to be mediated by Ca^2+^-permeable AMPA receptors (Sebe et al., 2017). Although excess Ca^2+^ entry *via* this receptor is likely to be the trigger for damage to the post-synaptic terminal, it remains unclear whether the oxidative stress involved in the later steps destroying the terminals. In contrast, the release of glutamate from IHCs is also controlled by the influx of Ca^2+^ to IHCs. It is worth investigating whether this Ca^2+^ influx could be reduced by a toughening noise which could increase Ca^2+^ buffering *via* calretinin, as proposed in a previous report (Alvarado et al., 2016).

Another potential mechanism underlying the cochlear protective effect of a toughening noise involves the cochlear efferent system. The efferent control is a significant mechanism involved in cochlear protection against noise-induced damage (Kujawa and Liberman, 1997; Rajan, 2000; Zheng et al., 2000; Le Prell et al., 2003; Darrow et al., 2007; Maison et al., 2013; Liberman et al., 2014), although the major role of this system is not to protect but rather to improve cochlear signal processing (Giraud et al., 1997; Christopher Kirk and Smith, 2003; Kumar and Vanaja, 2004; Yasin et al., 2014; Drga et al., 2016; Guinan, 2018; Marrufo-Pérez et al., 2018). However, most of these studies focused on the effect of middle efferents in protecting the OHC-based cochlear function. In studies of the toughening effect against noise, several have pointed to the role of the cochlear efferents (Yamasoba and Dolan, 1998; Attanasio et al., 1999; Canlon et al., 1999; Rajan, 2000; Niu and Canlon, 2002a). However, in all these studies, the status of the afferent cochlear synapses was not documented. Although there is no clear evidence of the role of the lateral efferents in the protective effect of toughening on afferent synapses, this possibility has been supported by several previously published studies. For example, it has been reported that selective removal of this innervation increases the vulnerability of the cochlea to noise (Darrow et al., 2007), although the study reporting this failed to examine synaptic status. Furthermore, in a recent report, the significant synaptic loss was seen in animals who had undergone deafferentation of the lateral olivocochlear innervation after being exposed to a noise that induced no synaptic loss in control animals (Maison et al., 2013). Also, the lateral efferent synapses in the cochlea release dopamine (DA), one of the potential neurotransmitters (Garrett et al., 2011; Maison et al., 2012; Toro et al., 2015; Valdés-Baizabal et al., 2015), and DA has been shown to reduce glutamate excitotoxicity (see review Lendvai et al., 2011). It is therefore worth evaluating whether a toughening noise enhances the release of DA. In still another study, tyrosine hydroxylase was found to be up-regulated in the lateral efferents to the IHC-SGN terminals by a preconditioning noise (81 dB SPL, 24 h), and this up-regulation was considered as the protective mechanism by the toughening noise (Niu and Canlon, 2002a). This is the only study we found so far that directly address the effect of toughening noise on lateral efferent innervation. However, since the synapse was not assessed, it remains unclear whether such enhancement protects the synapses from the damaging noise.

In the present study, we did not examine the potential impact of sex on the toughening effect in noise-induced synaptic loss. The gender impact on noise-induced synaptic damage has not yet been comprehensively investigated. However, in one recent study, no sex difference was found in the amount of synaptic loss caused by exposure to a 101 dB SPL and noise for 2 h in C57 mice, although a larger threshold shift was seen in male mice (Milon et al., 2018). Although the role of gender in the toughening effect has not yet been investigated, it is unlikely that this factor would play an important role in noise-induced synaptic damage.

Another limitation of the present study is the lack of detail change in synaptic morphology rather than simple synaptic counts. The detail on the synaptic size, shape and other pre- and postsynaptic structures, as well as the shape of IHCs and variation of such details across the groups, should shed light on the potential mechanisms of the toughening effect. Those issues should be further addressed in future studies.

In summary, the present study demonstrates that the toughening effect exists as a protective mechanism against noise-induced synapse loss. However, the suprathreshold auditory functions examined in this study were not well protected in the toughened animals in our toughening protocol, which may have induced minor, undetected damage to the synapses. The explored mechanisms underlying the toughening effect on the protection of OHCs and the loss of auditory sensitivity are, for the most part, not applicable to the protection against the synaptic loss. Further research is warranted to confirm the scope of the synaptic protective effect of toughening and its associated mechanisms.

## Data Availability Statement

The datasets generated for this study are available on request to the corresponding author.

## Ethics Statement

The animal study was reviewed and approved by Institutional Animal Care and Use Committee (IACUC) of the Affiliated Sixth People’s Hospital, Shanghai Jiao Tong University (permit number DWLL2017-0295).

## Author Contributions

LF and JW designed the research project. LF, ZZ, CL, HW, and YX performed the experiments. LF and ZZ analyzed the morphology data. JW, LF, and ZZ analyzed the functional data. LF, ZC, and ZZ drafted the manuscript. JW and SY edited and revised the manuscript and approved the final version of the manuscript.

## Conflict of Interest

s The authors declare that the research was conducted in the absence of any commercial or financial relationships that could be construed as a potential conflict of interest.

## Abbreviations

ABR: auditory brainstem response
AM: amplitude modulation
CAP: compound action potential
OHC: outer hair cell
IHC: inner hair cell
CtBP2: C-terminal binding protein 2
SGN: spiral ganglion neuron
TB: tone bursts.
